# Recurrence and influencing factors of moderate-to-severe atopic dermatitis after dupilumab withdrawal: a retrospective cohort analysis

**DOI:** 10.3389/fmed.2025.1585368

**Published:** 2025-08-13

**Authors:** Maohua Chen, Xinyu Wen, Jie Liu, Ge Yang, Qingqing Li, Zhiyuan Jiang, Xinjie Zhang, Zhen Cai, Lixia Zhang

**Affiliations:** ^1^Department of Plastic Surgery, Sichuan Provincial People’s Hospital, School of Medicine, University of Electronic Science and Technology of China, Chengdu, Sichuan, China; ^2^Institute of Dermatology and Venereology, Sichuan Provincial People’s Hospital, School of Medicine, University of Electronic Science and Technology of China, Chengdu, Sichuan, China; ^3^Department of Pharmacy, Sichuan Provincial People’s Hospital, University of Electronic Science and Technology of China, Chengdu, Sichuan, China; ^4^Department of Plastic Surgery, Sichuan Provincial People’s Hospital, Southwest Medical University, Luzhou, Sichuan, China

**Keywords:** atopic dermatitis, dupilumab, relapse on discontinuation, clinical remission, influencing factors

## Abstract

**Background:**

Atopic dermatitis (AD) is a chronic, recurrent, inflammatory skin disease. Although dupilumab has demonstrated favorable efficacy in the treatment of patients with moderate-to-severe atopic dermatitis, data on its recurrence after discontinuation remain limited.

**Objective:**

To explore the recurrence rate, time to recurrence, and factors influencing recurrence in patients with moderate-to-severe AD after discontinuing dupilumab, to bridge the existing knowledge gap and provide a reference for promoting long-term standardized management of the disease in AD patients to reduce AD recurrence.

**Methods:**

Patients with moderate-to-severe AD treated with dupilumab between January 2021 and December 2023 at Sichuan Provincial People’s Hospital were included. All patients started from the time of drug discontinuation, and baseline characteristics of patients were collected from all enrolled patients, and follow-up visits were conducted every 2 weeks after drug discontinuation utilizing telephone or medical records. Descriptive statistics summarized the relapse rate and time to relapse, and the Cox proportional hazards model was applied to determine the predictive factors of relapse after discontinuing dupilumab.

**Results:**

By the follow-up cut-off time, the median follow-up time was 49 weeks (24–85 weeks), and 141 AD patients were finally included in the statistical analysis. Of the 141 patients, 33 patients relapsed, with a relapse rate of 23.4% (95% CI, 16–30%), and the median time to relapse was 29 weeks. Predictors with a significant effect on recurrence included allergic conjunctivitis (HR = 7.912, 95% CI, 1.280–48.895, *p* = 0.026), duration of treatment <16 weeks (HR = 5.871, 95% CI, 2.154–16.003, *p* = 0.001), BMI ≥ 28 (HR = 5.653, 95% CI, 2.331–13.713, *p* < 0.001), male (HR = 5.634, 95% CI, 1.727–18.373, *p* = 0.004), and positive familial predisposition to allergy (HR = 3.438, 95% CI, 1.351–8.747, *p* = 0.01).

**Conclusion:**

The cumulative recurrence rate in 141 AD patients was 23.4%; the median time to recurrence in 33 AD recurrence patients was 29 weeks (22–59 weeks); comorbid allergic conjunctivitis, treatment duration shorter than 16 weeks, obesity, male patients, and positive familial predisposition to allergy were independent risk factors for AD recurrence. These findings confirm the disease characteristic of AD’s susceptibility to relapse and emphasize the need for individualized treatment, post-discontinuation monitoring, and long-term standardized management of AD patients with different risk factors for relapse.

## 1 Introduction

Atopic dermatitis (AD) is a chronic, relapsing, inflammatory skin disease that affects approximately 15–20% of children and 2–7% of adults worldwide, severely impacting the quality of life of millions of people (1, 2). Depending on the severity it can be categorized as mild, moderate, and severe (3). Because of its complex pathogenesis, diverse etiological factors, heterogeneity, and other disease characteristics, the course of the disease is often prolonged, and easy to recur, and treatment and long-term management of the disease is difficult. The short-term goals of AD treatment are mainly to effectively relieve itching, improve skin lesions, and improve quality of life, while the long-term goals are mainly to prevent and reduce AD recurrence, reduce or mitigate comorbidities, and achieve long-term control of AD. With the growing understanding of the disease, its treatment has progressed from broad-spectrum therapies such as glucocorticoids, calmodulin phosphatase inhibitors, and immunosuppressants to targeted biologics that target important inflammatory mediators. Dupilumab, a monoclonal antibody that blocks interleukin-4 and interleukin-13 signaling, works by blocking specific immune pathways involved in the pathogenesis of AD (4), and has demonstrated significant efficacy in improving patients’ symptoms, attenuating inflammatory responses, and increasing remission rates (5, 6), which represents a therapeutic breakthrough for moderate-to-severe AD. It also reduces the risk of asthma exacerbation in refractory AD patients with associated respiratory problems (7), and is therefore increasingly recommended globally as a first-line therapy for difficult-to-control or refractory cases (6).

Although the side effects of dupilumab are usually mild and transient, including injection-site reactions and occasional ocular symptoms such as conjunctivitis (5, 8–10), the efficacy and safety of long-term dupilumab use still needs more data support and close observation. Dupilumab controls symptoms while in use, but relapses may occur after discontinuation of use. The incidence of relapse varies in different populations with different disease exposures. How to choose the most appropriate treatment regimen for different populations, which can ensure sustained efficacy while minimizing the probability of disease recurrence, and thus reduce the burden of disease on patients has become a key issue that needs to be urgently resolved. Given the complexity and significant economic impact of lifelong adherence to treatment regimens (11–13), elucidating the likelihood of relapse and identifying predictive factors can greatly influence a patient’s long-term disease management strategy, including strategies to minimize relapse by developing a long-term maintenance treatment plan (through dose reductions, longer intervals between doses, and so on), and by personalizing the treatment plan for different patients.

The purpose of this paper is to analyze retrospectively the chart records of 141 patients with moderate-to-severe AD to study in depth the relapse of patients who discontinued dupilumab after clinical improvement or remission, to explore the associated risk factors leading to their relapse and to further investigate the temporal pattern of relapse, with a view to providing practical insights into the real-world outcomes of dupilumab discontinuation, thus contributing to the context of AD management in China. This will help to develop evidence-based guidelines for the prudent use and discontinuation of dupilumab in populations with different characteristics and provide direction for an effective response to the current challenge of AD relapse.

## 2 Methods

### 2.1 Study design and setting

Follow-up of patients with moderate-to-severe AD treated with dupilumab in the dermatology outpatient clinic of Sichuan Provincial People’s Hospital between January 1, 2021, and December 31, 2023, after discontinuation of the drug followed the Strengthening the Reporting of Observational Studies in Epidemiology (STROBE) guidelines. Patients began follow-up after discontinuation of medication, and follow-up observations were made every 2 weeks until recurrence of AD symptoms or cohort termination time (December 31, 2023). Using standard-of-care criteria to assess improvement or remission of disease symptoms following the recommendations of the Expert Guidelines for Systemic Medication Attainment in Moderate-to-Severe AD (14), that is, attainment of standardized treatment over 3 months includes a decrease in peak pruritus score of ≥4 points at 2 weeks, attainment of 50% lesion remission with an absolute peak pruritus score of ≤4 points at 4 weeks, and atopic dermatitis control tool (Atopic Dermatitis, Control Tool, ADCT) score of less than 7 points at 12 weeks and beyond. The study aimed to collect information on the recurrence of AD symptoms after discontinuation of dupilumab treatment in patients with moderate-to-severe AD whose clinical symptoms improved or resolved during treatment.

### 2.2 Participants

**Figure 1:**
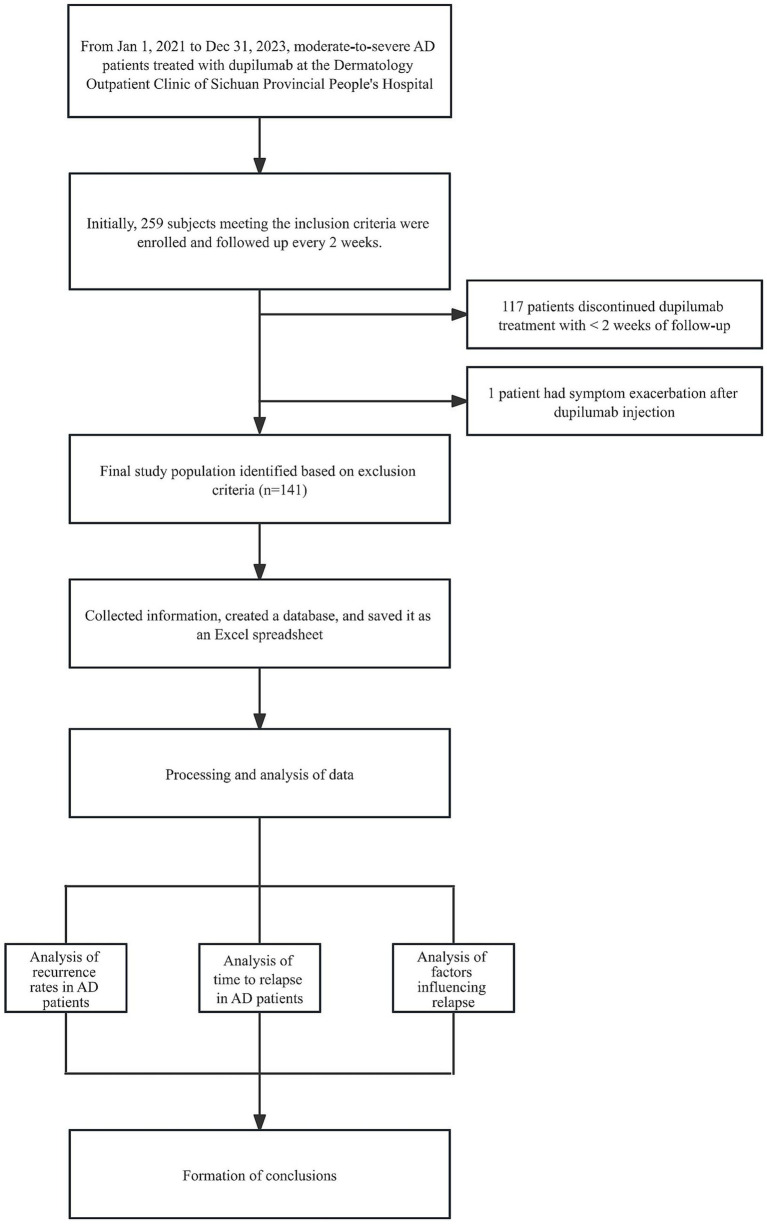
Study design and protocol. AD, atopic dermatitis.

Data were retrospectively collected from patients with moderate-to-severe AD who were seen in the dermatology outpatient clinic of the Sichuan Provincial People’s Hospital from January 1, 2021, to December 31, 2023 and were treated with dupilumab (Sanofi, France). Inclusion criteria: 1. Meet the American Academy of Dermatology/European Society of Dermatology and Venereology criteria (15) AD diagnostic criteria; 2. Discontinuation of dupilumab for any reason after experiencing a predetermined period of clinical improvement or remission; 3. Regular medication and course of treatment ≥ 4 weeks; 4. Received standard induction dose (600 mg, body weight ≥30 kg/300 mg, body weight <30 kg) and maintenance dose [300 mg, q2w (body weight ≥30 kg)/300 mg, q3w (body weight <30 kg)] treatments; 5. Past medical records and follow-up information were complete; 6. For those who need to allow the combination of hormones, calmodulin phosphatase inhibitors, and Januskinase (JAK) inhibitors during treatment based on a comprehensive evaluation of the disease. Exclusion criteria: 1. patients with severe functional insufficiency of heart, liver, kidney, and other important organs and/or diabetes mellitus; 2. Patients with an allergic reaction to the components and excipients of Dupilumab, or the condition worsened or new complications appeared after the use of Dupilumab; 3. Follow-up time after stopping the drug <2 weeks. A total of 141 patients with moderate-to-severe AD were finally included (Figure 1).

### 2.3 Data collection

Patient information was collected by retrieving and systematically reviewing the electronic medical records of included patients and by telephone follow-up (data anonymization, patient informed consent waiver). The data included patients’ general condition (gender, age, body mass index), duration of disease, comorbidities, family history, blood-related markers, dupilumab monotherapy dosing regimen and duration, co-administration, history of previous medications, time of discontinuation of dupilumab monotherapy and time of first flare-ups, and EASI (Eczema Area and Severity Index) and SCORAD (Score of Atopic Dermatitis) before treatment, NRS (numeric pain rating scale), DLQI (dermatologic quality of life index) and ADCT (atopic dermatitis control tool) before and after treatment. Follow-up visits were conducted every 2 weeks after discontinuation of the drug, and follow-up data were obtained from electronic medical record records and/or direct patient contact, with a data collection deadline of December 31, 2023.

### 2.4 Outcome measurement

The primary observation was the recurrence rate of AD after discontinuation of dupilumab treatment per 100 patients-year follow-up. Secondary observations included the time interval between discontinuation of dupilumab and first recurrence, and the identification of associated potential risk factors, such as age, sex, family history, and comorbidities. As the criteria for AD relapse are still not uniform, the present study adopted the European Task Force on Atopic Dermatitis (ETFAD) definition of AD relapse as “an acute, clinically significant worsening of the signs and symptoms of AD that requires therapeutic intervention” (16), with the time point of relapse being determined by a patient-initiated report of worsening, followed by an experienced dermatologic clinical. The time point of relapse is determined by the patient’s voluntary report of deterioration and confirmation of relapse by an experienced dermatologic clinician. The relapse interval was the time interval between the point at which the patient’s last dose of dupilumab was administered and the point at which the patient needed to be reintroduced to the therapeutic intervention.

### 2.5 Statistical analysis

Descriptive statistics were used to summarize the characteristics of the study population. Kaplan–Meier survival analysis was used to plot survival curves and estimate recurrence-free survival rates, Cox proportional risk regression models were constructed to assess the association between candidate risk factors and recurrence and to calculate hazard ratios (HR) and 95% confidence intervals (95% CI) for AD recurrence. Multivariate analysis was performed to control for confounding variables.

Measured variables are expressed as mean and standard deviation or median and interquartile spacing (IQR), and count data are expressed as cases (%). The 95% confidence intervals (CI) were calculated using the Clopper-Pearson exact method. The cumulative incidence of AD recurrence was estimated using COX regression. Subgroup analyses were performed to assess the association between AD recurrence and several predefined variables: gender, age ≥60 years, body mass index ≥28, duration of disease ≥10 years, duration of dupilumab treatment before discontinuation (<16 weeks vs. ≥16 weeks), familial allergic tendency, and corticosteroid treatment. Statistical analyses were performed using SPSS (IBM SPSS Statistics for IOS, version 26.0, IBM Corporation) and graphs were generated using GraphPad Prism 9.0. Demographic data and clinical characteristics were analyzed by descriptive analysis. The significance test level was *p* < 0.05.

### 2.6 Ethics statement

The study protocol was approved by the Institutional Review Board (IRB) of the People’s Hospital of Sichuan Province (approval no. 2022-327). Due to the retrospective nature of this cohort study, where patient data were anonymized and retrospectively analyzed from available medical records, the institutional review board (IRB) waived informed consent for participation in the study. For cases requiring additional information to be collected or obtained specifically for this study, written or verbal informed consent was obtained following protocols approved by the Institutional Review Board. Patient confidentiality was maintained throughout the study by the Health Insurance Portability and Accountability Act (HIPAA) and local ethical standards.

## 3 Results

After the exclusion of 117 patients with less than 2 weeks of follow-up after discontinuation of dupilumab therapy from the 259 AD patients with dupilumab injections who were included, 1 patient experienced a worsening of symptoms after dupilumab injections, and 141 patients were finally included (Figure 1), Patients had a mean [SD] age of 37.3 [20.7] years, 57.4% were male, and at a median follow-up of 49 weeks after discontinuation of medication (IQR, 24–85 weeks), 33 patients (23.4%; 95% CI, 16–30%) experienced AD recurrence. One patient was observed to die from serious complications during discontinuation follow-up, and there were no fatal cases of AD recurrence (Table 1).

**Table 1:** Comparison of baseline characteristics and clinical characteristics between atopic dermatitis patients with and without recurrence.

	Total (*n* = 141)	Recurrence (*n* = 33) (%)	No recurrence (*n* = 108)	*p* value
Age≥60 years, *n* (%)	28 (19.9)	11 (33.3)	17 (15.7)	0.846
Male, *n* (%)	81 (57.4)	29 (87.9)	52 (48.1)	0.005
BMI ≥ 28, mean (SD)	22.16 (3.6)	22.28 (9.0)	22.10 (13.6)	0.001
Course of disease, mean (SD), y	9.76 (7.9)	9.8 (61.6)	9.8 (61.8)	0.349
Comorbidities				
Allergic rhinitis, *n* (%)	48 (34.0)	11 (33.3)	37 (34.3)	0.913
Asthma, *n* (%)	18 (12.8)	5 (15.2)	13 (12.0)	0.878
Nettle rash, *n* (%)	17 (12.1)	8 (24.2)	9 (8.3)	0.918
Allergic conjunctivitis, *n* (%)	4 (2.8)	2 (6.1)	2 (1.9)	0.023
Chronic bronchitis, *n* (%)	8 (5.7)	3 (9.1)	5 (4.6)	0.344
Risk factor for AD				
Familial predisposition of allergy, *n* (%)	49 (34.8)	25 (75.8)	24 (22.2)	0.008
Corticosteroid therapy, *n* (%)	67 (47.5)	26 (78.8)	41 (38.0)	0.981
Dupilumab treatment duration, ≤16 W, *n* (%)	55 (39.0)	15 (45.5)	40 (37.3)	0.001
Dupilumab treatment duration, median (IQR), week	24 (12 ~ 62.4)	24 (12 ~ 34.1)	25.9 (13.5 ~ 89.5)	NA
Follow up, median (IQR), W	49 (24 ~ 85)	29 (22 ~ 59)	53.5 (30.5 ~ 87)	NA
Time from discontinuation of dupilumab to recurrence, median (IQR), W	NA	29 (22 ~ 59)	NA	NA

AD, atopic dermatitis; BMI, body mass index (calculated as weight in kilograms divided by height in meters squared); IQR: interquartile range. ^a^Data are presented as number/total number of patients (percentage) unless otherwise indicated.

**Figure 2:**
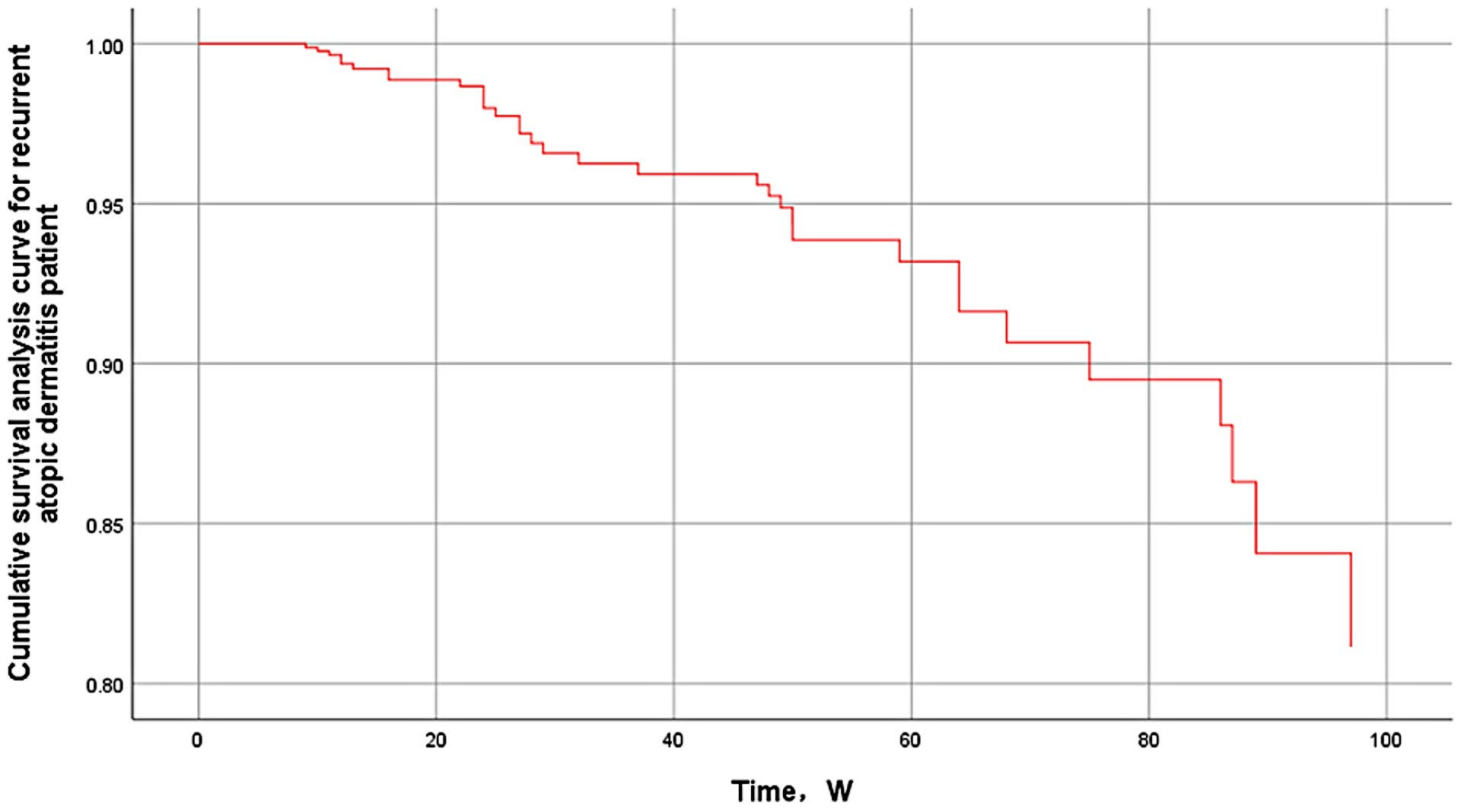
Cumulative survival analysis curve for recurrent atopic dermatitis.

Of particular note, the AD population with allergic conjunctivitis had the highest rate of recurrence, with a hazard ratio (HR) of 7.912 (95% CI 1.280–48.895, *p* = 0.026), followed by those who had been treated with dupilumab for <16 weeks (HR = 5.871, 95% CI 2.154–16.003, *p* = 0.001), and those who had a BMI ≥ 28 (HR = 5.653, 95% CI 2.331–13.713, *p* < 0.001), male (HR = 5.634, 95% CI 1.727–18.373, *p* = 0.004), and positive familial predisposition to allergy (HR = 3.438, 95% CI 1.351–8.747, *p* = 0.01). Although modest increases were observed in urticaria (HR = 1.035), and allergic rhinitis (HR = 1.023), none of these factors reached statistical significance in this study. Asthma (HR = 0.935), previous corticosteroid treatment (HR = 0.880), age ≥60 (HR = 0.763), duration of disease ≥10 years (HR = 0.761), and comorbid chronic bronchitis (HR = 0.416) were negatively associated with risk (see Figure 2).

**Figure 3:**
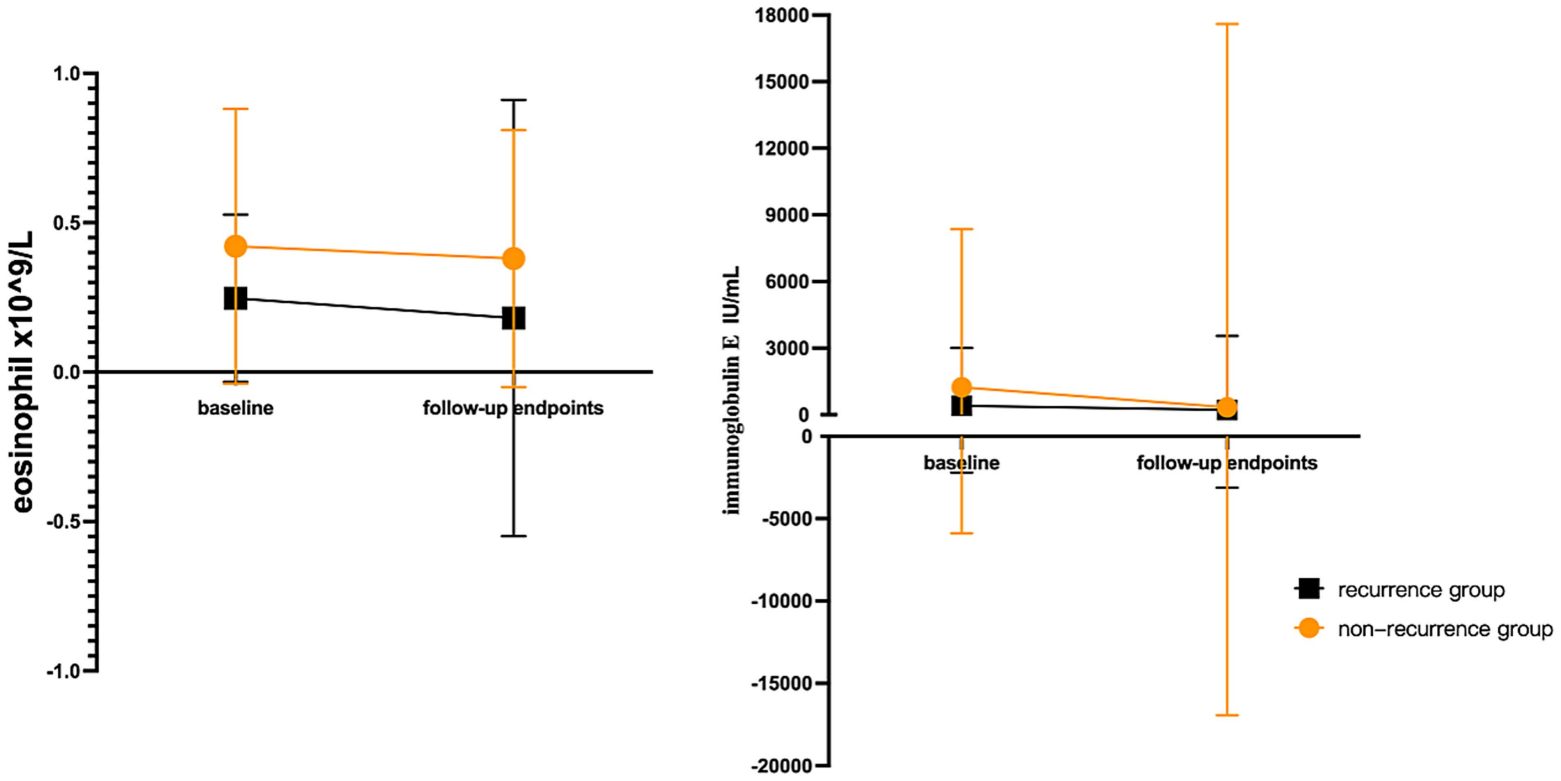
The changing trend of eosinophils and IgE laboratory results of AD patients at baseline and follow-up endpoints. The magnitude of EOS changes was greater and IgE changes were smaller in the recurrent population. EOS: eosinophil; IgE: immunoglobulin E.

The study showed significant changes in eosinophils (EOS) and relatively small fluctuations in immunoglobulin E (IgE) in the relapsed population (Figure 3).

## 4 Discussion

Despite the proven efficacy of dupilumab in the treatment of moderate-to-severe AD, the evidence base for dupilumab in the Chinese population is still relatively scant and there are few reports in the literature of relapses after discontinuation of dupilumab treatment in patients with AD. Therefore, we conducted an in-depth study of relapse and factors affecting relapse after discontinuation of dupilumab in Chinese patients to fill this important knowledge gap.

First, our study observed that during the follow-up of 141 patients with moderate-to-severe AD, 33 patients experienced a relapse of AD after discontinuing dupilumab therapy, with a relapse rate of 23.4%, a median time to relapse of 29 weeks (22 to 59 weeks), and a median follow-up time of 49 weeks (24 to 85 weeks). A retrospective study of relapse after discontinuation in patients with moderate-to-severe adolescent AD treated with dupilumab (17) observed that 54. 8% (17/31) of 31 patients who discontinued the drug experienced relapse during a follow-up of at least 11 months, with a median time to relapse of 270 days, which is a higher rate of relapse but a longer median time to relapse in comparison to the present study, which may be attributed to the following reasons. First, the study did not specify discontinuation criteria, and some patients did not meet the clinical criteria for discontinuation (2 due to financial burden and 28 due to improvement in skin condition), which may have led to a higher relapse rate. Second, in the choice of treatment regimen, patients weighing <60 kg received an initial dose of 400 mg, followed by 200 mg every 2 weeks for at least 8 weeks, which was a higher dose and a longer maintenance period than that used in the present study, and may account for the longer interval between relapses. In addition, the study was conducted on a population of adolescents 12 ≤ age < 18, which had different predictors of recurrence than the present study population, such as allergic conjunctivitis, BMI, and duration of treatment. Due to the diversity of AD recurrence factors, it is not possible to speculate at this time whether the combined effect will result in a statistical difference.

Second, our findings also shed light on several important factors that contribute to the increased risk of relapse in patients with moderate-to-severe AD after discontinuing treatment with dupilumab. Notably, allergic conjunctivitis was the main risk factor with a high hazard ratio of 7.912 (95% CI, 1.280–48.895, *p* = 0.026), followed by treatment duration of less than 16 weeks (HR = 5.871, 95% CI, 2.154–16.003, *p* = 0.001), male (HR = 5.634, 95% CI, the1.727–18.373, *p* = 0.004), BMI ≥ 28 (HR = 5.653, 95% CI, 2.331–13.713, *p* < 0.001) and positive familial predisposition to atopy (HR = 3.438, 95% CI, 1.351–8.747, *p* = 0.01).

At the same time, this paper also provides a preliminary mechanistic discussion of the causes of recurrence due to the above recurrence risk factors. First, comorbid allergic conjunctivitis, a retrospective study (18) noted a correlation between the male sex (HR, 2.34; 95% CI, 1.14–4.78; *p* = 0.02) and propensity to allergic conjunctivitis (HR, 2.61; 95% CI, 1.37–5.00; *p* = 0.004) and reduced drug survival during dupilumab treatment, Also, patients with allergic conjunctivitis may be at higher risk for ocular-related adverse events, which may affect their continued use of dupilumab therapy and therapeutic efficacy in AD. In addition, allergic conjunctivitis, as one of the co-morbidities in the atopic process with AD as the “starting point,” is also one of the common complications of AD treated with Dupilumab, and the presence of atopic co-morbidities may reflect the subclinical state of the disease as well as immune imbalance due to activation of the systemic type 2 inflammatory cascade (19), which may reduce the responsiveness to dupilumab treatment.

Moreover, treatment duration is less than 16 weeks, first, probably since AD disease itself involves multiple developmental stages, including asymptomatic preclinical stages (20), activation of cutaneous innate immunity, initiation of adaptive immune responses, and subsequent expansion of immune responses (21). Secondly, it may involve “tissue memory” (22) in disease modification, the long-term effects of chronic inflammation on cells and tissues, where the body’s response to inflammation does not stop even after the initial inflammatory stimulus has been eliminated, and this inflammatory process persists in the non-lesional skin of moderate-to-severe AD (23–27), triggering a relapse or comorbidity of AD. Short-term induction therapy may be only visible relief of skin lesion symptoms, while the disease remains in a subclinical state that needs to be maintained for a long time to control the subclinical state and progressively achieve clinical remission without medication, therefore, too short a duration of treatment may be a risk factor for disease recurrence (28–30). A phase III trial (31) investigating the long-term efficacy and safety of dupilumab in the treatment of adolescents with moderate-to-severe atopic dermatitis observed that 30 patients started to discontinue the drug and were monitored for relapse after 12 consecutive weeks of maintaining IGA 0/1 (complete or almost complete clearance of skin lesions) after week 40 (i.e., a total of 52 weeks of treatment prior to discontinuation), and that 17 patients who relapsed had a median time to relapse of 17.5 weeks (2 to 64 weeks). The 33 relapsed patients in this study were treated for a median of 24 weeks, and the median time to relapse was 29 weeks (22 to 59 weeks). We hypothesise that the time taken to achieve clinical remission with dupilumab may influence the late recurrence of the disease, as the immune response to drug modulation varies among individuals, affecting the likelihood of the potential inflammation to “return” in subclinical organisms and the time to recurrence after discontinuation of the drug.

Thirdly, male gender and BMI ≥ 28 were observed to be higher in a Finnish cross-sectional study (32) in patients with severe AD, while possible determinants of disease severity included being male (*p* = 0.002, 95% CI 0.000–0.11) and having a high BMI (*p* < 0.000, 95% CI 0.000–0.011). In addition, Holm et al. (33) similarly observed a higher proportion of men with severe AD in a similar cross-sectional Danish study. Therefore, a correlation between male gender and AD disease severity can be tentatively inferred, which may lead to poor efficacy of dupilumab in patients with this risk factor, and a delayed inflammatory process leading to relapse. A prospective observational real-world study (32) assessed the psychological outcomes of 3 years of treatment with dupilumab for moderate-to-severe AD, in which women showed more significant improvements than men in certain mental and physical health outcomes, and in which aggravated and fluctuating moods could exacerbate the disease symptoms of AD, which can lead to AD relapse. And, also real-world studies (30) have observed female gender (34, 35) and BMI < 24 as factors for favorable response to dupilumab. A retrospective study (31) also observed lower susceptibility to allergic conjunctivitis (HR, 2.34; 95% CI, 1.14–4.78; *p* = 0.02) and susceptibility to allergic conjunctivitis (HR, 2.61; 95% CI, 1.37–5.00; *p* = 0.004) in males (HR, 2.61; 95% CI, 1.37–5.00; *p* = 0.004) with dupilumab which is associated with drug survival and may be associated with an increased risk of AD recurrence. Previous studies (36) have found that obesity is not only associated with the severity of AD, and a retrospective study (37) observed that a high BMI was associated with a low response rate to early treatment with dupilumab, suggesting that obesity is not only associated with the onset of AD, but may also affect its therapeutic efficacy, possibly due to the fact that higher doses of the drug are required to achieve steady-state concentrations in obese patients due to increased body weight and resulting in increased drug distribution volume. Obesity may reduce early drug exposure, leading to delayed efficacy (38). Obesity and metabolism can also affect the immune system, with obese mouse models of atopic dermatitis (39) showing a 2-4-fold increase in lesion thickness compared to leaner models. In addition, obesity may alter the body’s immune microenvironment from a classical disease dominated by type 2 T helper cells (TH2) to a more severe disease dominated by TH17 inflammation, leading to therapeutic target shifting by reprogramming the T cell response pattern, resulting in dupilumab showing exacerbated epidermal hyperplasia and TH17 inflammation in obese mice.

In addition to the above recurrence factors explored in this study, a previous retrospective study (40) comparing the risk of recurrence between patients who achieved a 75% improvement in EASI on their last injection of dupilumab and those who did not, demonstrated that the risk of recurrence was significantly lower in EASI 75 achievers than in non-achievers, with 6-month recurrence rates of 47.4 and 64.3%, respectively, suggesting disease recurrence may be related to early treatment response. In addition, a study of AD in the elderly (41) also noted that disease severity and early response to treatment may be associated with disease recurrence. Of interest, studies exploring the risk of relapse after discontinuation of dupilumab in pediatric populations (40) similarly noted that factors such as achievement of EASI75 at the time of the last dupilumab injection, early response to treatment, disease severity, and scores at baseline were all associated with the risk of relapse in AD. It can be seen that there are a variety of factors that contribute to AD disease recurrence, and the response to early treatment with dupilumab and the severity of the disease itself may also influence the risk of recurrence to some extent.

This emphasizes the complex interplay of multiple risk factors that influence relapse and the need to develop specific and personalized treatment regimens for patients with risk factors to reduce the risk of relapse after discontinuation. However, further prospective studies are needed to elucidate the exact impact of these risk factors on the relapse rate after discontinuation of dupilumab.

Dupilumab has been shown to have excellent efficacy in moderate-to-severe AD, providing effective relief of itching and skin lesions within four to six weeks of use (42), but because of the heterogeneity and instability of AD disease, choosing the appropriate maintenance dose of dupilumab after discontinuing treatment may minimize the rate of relapse. Whereas the customization of the maintenance dose depends on the number of risk factors to which the patient is exposed, this process is inherently dependent on the professional judgment of experienced dermatologists and their flexibility in the application of tools such as the Atopic Dermatitis Control Tool (43). The Expert Consensus on Stratified Individualized Systemic Therapy for Moderate-to-Severe Atopic Dermatitis (44) suggests that patients with moderate-to-severe or relapsing AD should be transitioned to “active maintenance therapy” after control of lesions, with a gradual reduction in dosage and longer dosing intervals, to achieve the goal of long-term maintenance therapy. It is recommended that when using anti-IL-4Rα monoclonal antibody in the treatment of AD, the drug should be maintained every 2–4 weeks for a long period, with a gradual extension of the dosing interval after reaching EASI 90 or IGA 0/1. Early therapeutic intervention through maintenance dosing is effective in preventing the worsening of mild symptoms and escalation to more severe manifestations, which may reduce recurrence rates of AD to a greater extent and reduce the need for intensive treatment regimens and their associated adverse effects. In conclusion, the active administration of maintenance doses in the management of AD disease can provide a range of benefits. However, further high-quality, real-world research data are needed to support the precise efficacy of long-term disease management regimens such as extended dupilumab maintenance therapy and extended dosing intervals, as well as to explore the development of specific regimens.

In addition to early intervention for AD relapse, we are equally concerned about the disease status of patients who have relapsed. Subsequent management and treatment of these relapsed patients typically include reassessment of the disease, possible reinitiation of dupilumab therapy, or transition to other alternative treatment regimens, with close monitoring to assess the efficacy of new interventions. In our cohort of relapsed patients, we observed that restarting dupilumab treatment remained effective in controlling disease recurrence. In the future, further follow-up could be conducted to investigate the efficiency and long-term efficacy of dupilumab in AD relapse patients with different risk factors.

Our study has several noteworthy strengths. First, this study represents the broadest sample of AD patients in Sichuan, China, providing valuable information to understand the epidemiology of the region. In addition, this study included both child and adult participants, which is consistent with the likely age distribution of the entire AD population in China, thus increasing the ecological validity of the findings. Finally, our study is expected to identify modifiable factors leading to AD relapse and provide insight into the risk profiles associated with relapse at different stages of the disease. However, our study has several limitations. Firstly, this is a retrospective study in the nature of clinical data and is therefore susceptible to information bias, recall bias, and detection bias, which are difficult to avoid despite our efforts to cross-validate through follow-up and medical records. During the study, we controlled for bias by double-checking and an independent review by 2 senior dermatologists to minimize bias. Second, this study only included partial data from Sichuan Province, China, which lacks generalizability to a wider population, due to poor patient compliance, incomplete data resulting in a limited sample size, and medical disparities in the management of atopic dermatitis due to differences in racial backgrounds, environmental exposures, social determinants, and education levels. Third, the limited sample size and subtle trends in EOS and IgE preclude definitive conclusions about these biomarkers. In future research, we plan to expand the sample size by collaborating with multiple institutions across different regions. This multi-center approach will enhance statistical power and improve the generalizability of our results. Prospective studies will also be considered to better control biases and increase the credibility of our findings.

## 5 Conclusion

In conclusion, this study confirms the disease characteristic of AD recurrence and emphasizes the necessity of individualized treatment, post-discontinuation monitoring, and long-term standardized management of AD patients with different risk factors for recurrence to minimize recurrence and optimize the overall health of the patients, as well as deepening the exploration of the determinants of AD recurrence and its mechanisms after discontinuation of dupilumab.

## Data Availability

The raw data supporting the conclusions of this article will be made available by the authors, without undue reservation.
